# Supplementary material for: Children’s attitudes towards animals are similar across suburban, exurban, and rural areas
Source: PeerJ. 2019 Jul 23;7:e7328. doi: 10.7717/peerj.7328 (PMC6659664; doi:10.7717/peerj.7328)
Supplement: Supplemental Information 6 [file peerj-07-7328-s006.pdf]

Date:________________________ School:___________________________
Class:_________________________ Teacher:__________________________

Q1. List four animals that you find most scary.

1.
2.
3.
4.

Q2. List four animals that you like the most.

1.
2.
3.
4.

Q3. Rank your top five favorite animals from the following list in order from your favorite (1) to your least favorite (5):

_______ kangaroo	_______ raccoon
_______ zebra	_______ skunk
_______ bobcat	_______ wolf
_______ lion	_______ monkey
_______ coyote	_______ fox
_______ deer	_______ bat
_______ rabbit	_______ hedgehog
_______ panda	_______ bear
_______ opossum	_______ squirrel
_______ rhinoceros	_______ whale

Are you a boy or a girl? ______________
What grade are you in? ______________
What is your ethnicity? (please check all that apply)
___Asian
___Black
___Hispanic or Latino
___Native American
___White
___Other

Do you hunt? **Yes** **No**

Does anyone in your family hunt? **Yes** **No**

Thank you for your participation!
